# Polyneuritis cranialis following actinomycosis of a wisdom tooth: a case report

**DOI:** 10.1186/s13256-025-05654-9

**Published:** 2025-11-19

**Authors:** Anna Maria Welzel, Mariya Kondova, Norbert Pfeiffer, Joanna Wasielica-Poslednik

**Affiliations:** 1 Department of Ophthalmology, University Medical Center of the Johannes Gutenberg-University Mainz, Langenbeckstr. 1, 55131 Mainz, Germany; 2 Department of Neuroradiology, University Medical Center of the Johannes Gutenberg-University Mainz, Mainz, Germany

**Keywords:** Neurotrophic keratopathy, Polyneuritis cranialis, Actinomycosis, Corneal ulcer

## Abstract

**Background:**

Polyneuritis cranialis is a rare condition than can be caused by various diseases. We report the first case of multiple cranial nerve paralysis resulting in neurotrophic keratopathy with corneal ulceration as a sequel of polyneuritis cranialis due to actinomycosis.

**Case presentation:**

An 84-year-old German woman developed corneal ulceration with a high risk of perforation because of neurotrophic keratopathy due to polyneuritis cranialis. Actinomycosis of a wisdom tooth causing polyneuritis cranialis was detected on computed tomography and magnetic resonance imaging and was treated with systemic antibiotics. Penetrating keratoplasty with simultaneous amniotic membrane transplantation was performed successfully after conservative treatment failure.

**Conclusions:**

To the best of our knowledge, we report on the first case of multiple cranial nerve failure due to actinomycosis and requiring ophthalmological intervention.

## Background

Neurotrophic keratopathy (NK) is a rare degenerative corneal disease (estimated prevalence less than 5/10,000) that is defined by the reduction or absence of corneal sensation due to impairment of trigeminal nerve (V) [1]. A reduced tear film production, no eyelid closure reflex, and consequent epithelial breakdown expose corneal stroma, turning it vulnerable to environmental stress and enzymatic degradation [1, 2]. Different conditions such as herpes simplex or zoster keratitis, chemical burns or previous corneal injuries, or surgery, which disrupt corneal innervation can result in a NK [3]. NK can be classified into three clinical stages: Stage 1, with punctate keratopathy and epithelial irregularity; Stage 2, with persistent epithelial defects; and Stage 3, with a corneal ulcer and melting, which can cause perforation [3].

Exposure keratopathy (EK) is characterized by dryness of the cornea and is mainly caused by lagophthalmos or proptosis and therefore incomplete blinking. Corneal desensitization or movement disorders, for example, Parkinson disease, can predispose a patient to a decreased blinking frequency and therefore increased exposure of the cornea. EK is significantly more common than NK and can also lead to epithelial defects and ulcers [4].

The combination of NK and EK can be a result of diverse underlying conditions such as cranial nerve paralysis of the trigeminal or facial nerve. Multiple nerve palsies mostly result from tumors, vascular diseases, trauma, or infections, followed by more uncommon causes such as surgical complications, polyneuropathies by diabetes mellitus, or Guillain–Barré syndrome [5].

We present a case report of multiple cranial nerve paralysis resulting in EK and NK with corneal ulceration as a sequel of polyneuritis cranialis due to actinomycosis of a wisdom tooth. Penetrating keratoplasty with amnion membrane transplantation was performed successfully after conservative treatment failure.

## Case presentation

An 84-year-old German woman was referred for consultation to the department of ophthalmology in July 2022. Recently, she had noticed an increasing redness and vision loss of the left eye. She expressed additional symptoms progressing since 2 years ago.

Her first symptom was hypesthesia surrounding the left eye followed by double vision and a hanging eye lid and decreased taste perception. Other pre-existing conditions were autoimmune hepatitis treated with prednisolone and azathioprine.

**Fig. 1:**
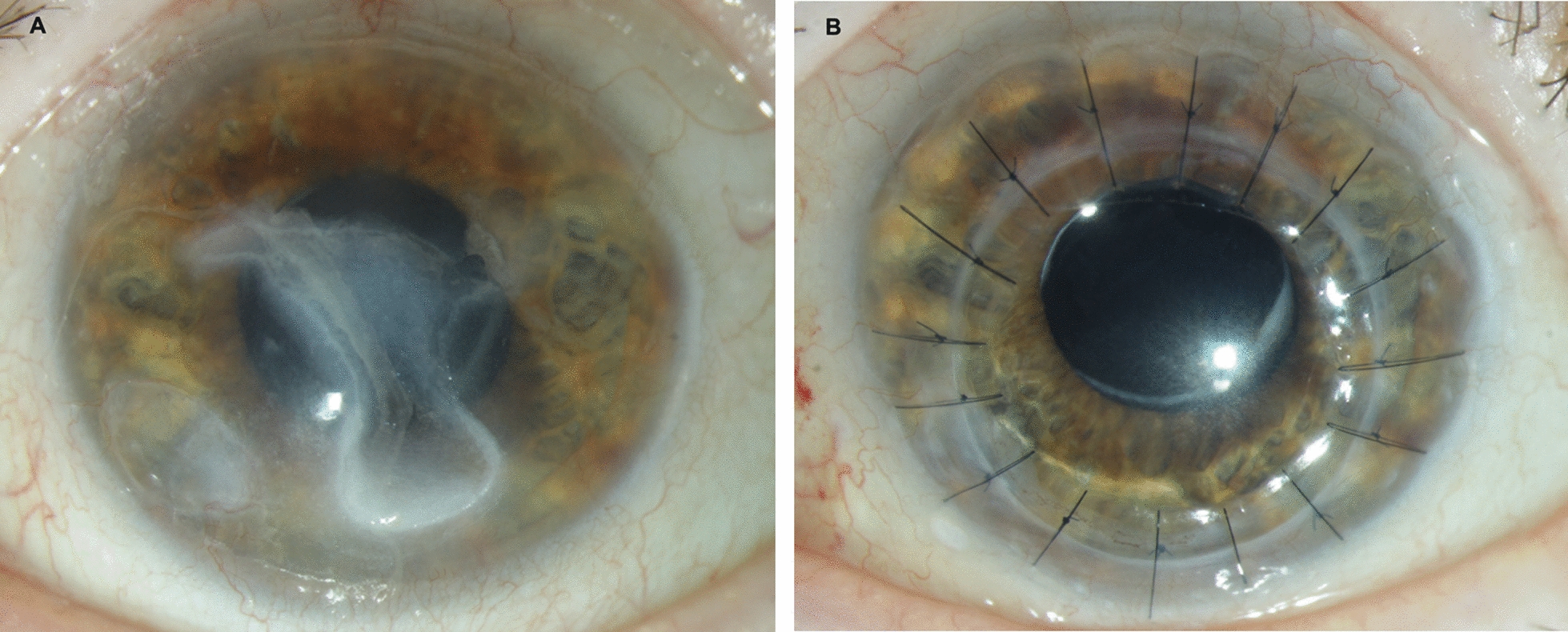
**a** A slit lamp photograph of the patient’s left eye showing a central corneal ulcer next to a cystic-appearing corneal defect close to the limbus. **b** A slit lamp photograph of the patient’s left eye 5 months after the last surgery showing a clear corneal graft

The clinical examination revealed a complete facial paralysis including ptosis, complete ophthalmoplegia, and an enlarged, rigid pupil (cranial nerve failure III–VII). Despite the ptosis we noticed a lagophthalmos of 1–2 mm. The corneal sensation was absent. A central corneal ulcer of 5.2 × 3.1 mm was located next to a cystic-appearing corneal defect (2.2 × 1.5 mm) (Fig. 1a). The central corneal thickness within the ulcer was 379 µm, measured by optical coherence tomography (OCT).

Her visual acuity was hand movements in the left eye and 20/20 in the right eye. The right eye was asymptomatic with a normal exam.

Under conservative treatment (artificial tears, topical dexamethasone, antibiotics, and a bandage contact lens) the corneal thickness within the ulcer reduced rapidly to 218 µm. Due to a high risk of perforation, a penetrating keratoplasty with simultaneous amniotic membrane transplantation was performed, without any complications. Furthermore, we injected botulinum toxin in the upper eyelid to minimize her lagophthalmos. The cornea has remained stable over 8 months since the last surgery (Fig. 1b).

**Fig. 2:**
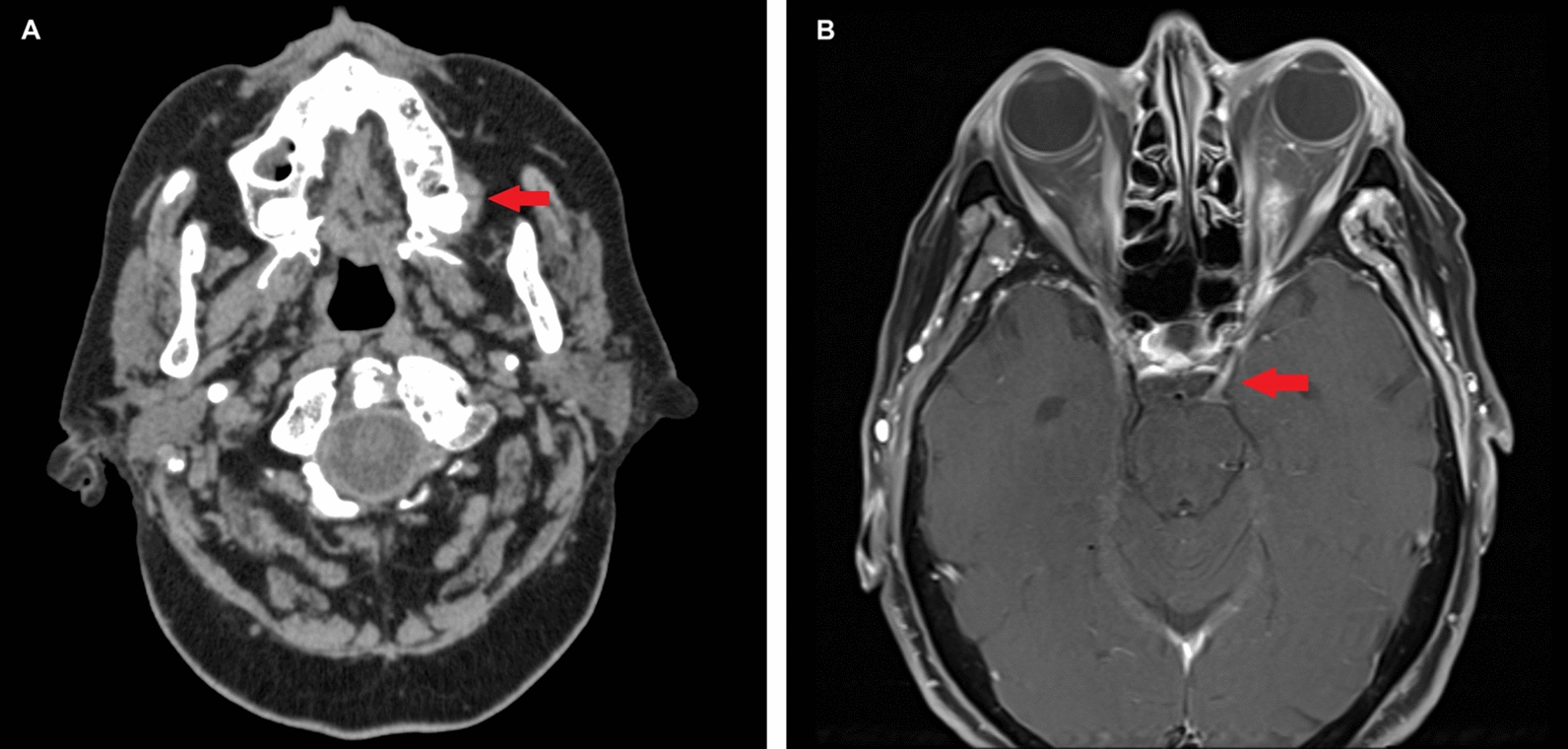
**a** Computed tomography scan: wisdom tooth with surrounding inflammation of the masticator space highlighted with red arrow. **b** Magnetic resonance imaging scan: exemplary oculomotor nerve with enhancement of contrast agent highlighted with red arrow

The cause of the cranial nerve failure resulting in NK remained unclear until a cranial computed tomography (CT) revealed a wisdom tooth with surrounding inflammation of the masticator space (Fig. 2a). We detected infiltration of the foramina ovale and rotundum, orbital apex, pterygopalatine, and infratemporal fossa following magnetic resonance imaging (MRI). With the exception of the facial nerve, all paralyzed nerves traverse outlined anatomic structures, and an enhancement of contrast agent was visible (Fig. 2b). Osteotomy and histopathological examination of the wisdom tooth revealed a population of actinomycosis with active affection. Reduction of her systemic immunosuppressants as far as her autoimmune hepatitis allowed and systemic antibiotic therapy with ampicillin/sulbactan, ertapenem, and moxifloxacin stopped the neurological deficits from progressing any further, but existing deficits have never recovered.

## Discussion and conclusion

To our knowledge, there is no case report on polyneuritis cranialis due to actinomycosis colonization with ophthalmologic consequences. Cranial nerve failure due to actinomycosis has been very rarely reported. Two cases illustrate a single nerve failure, and only in one report were multiple cranial nerves affected (glossopharyngeus, vagus, and hypoglossus [IX, X, and XII]) [6–8].

Actinomyces species bacteria are a common constituent of the gastrointestinal flora and can cause rare and chronic actinomycosis often causally related to the teeth in immunosuppressed patients [9, 10]. Actinomycosis can mimic a variety of infectious, inflammatory, or even malignant diseases, making a fast diagnosis challenging. Cervicofacial actinomycosis mostly affects facial soft tissues such as cheeks or chin. Symptoms vary from painful to painless swellings, fever, loose dentures, and spontaneous drainage of purulent yellowish thick exsudate [10]. Our patient denied such conditions and had no background of recent dental surgery. Actinomycosis, being uncommon itself, appeared atypically.

Infections originating in the maxillary teeth are known to spread along various pathways. Obayashi *et al*. [11] reported masticator space changes to be predominantly seen in patients with infection caused by molars in CT scans, which matches our case report. Even uncommon systemic infections, such as tetanus (*Clostridium tetani*) were reported to originate from odontogenic infections [12].

Impairment of the trigeminal nerve led to development of NK with corneal melting. The therapy of NK regardless of the underlying pathomechanism is challenging, with various possible treatment options, which include local application of artificial tears, autologous serum, anti-inflammatories, or the usage of bandage contact lens. Corneal ulceration can be addressed with amniotic membrane transplantation, but perforation or a high risk of it may unavoidably lead to penetrating keratoplasty or even sclerokeratoplasty.

In our case we performed a ptosis induction by injecting botulinum toxin in the upper eye lid. The imminent loss of the eyeball was averted with penetrating keratoplasty and amniotic membrane transplantation. A close monitoring is necessary to detect and treat new epithelial defects, which may occur in the future. Furthermore, a close neurological follow-up is necessary to monitor neurological deficits and to be able to intervene as early as possible in the event of renewed progression.

To conclude, actinomycosis is a rare cause for neurological deficits in particular for cranial nerve failure. Interdisciplinary cooperation was essential to detect its origin and made an intervention possible. From an ophthalmological point of view, the combination of NK and EK regardless of the underlying pathomechanism is a challenging clinical picture with various possible treatment options.

## Data Availability

Please contact the author for data requests.
